# The Vaccination Cases at Westford

**Published:** 1860-05

**Authors:** 


### THE VACCINATION CASES AT WESTFORD.

Much excitement has lately been caused in Westford and its vicinity, by the serious effects which have followed several cases of vaccinatien in that town. From the report made at the last meeting of the Boston Society for Medical Improvement, it appears that the matter used consisted of crusts, taken from healthy persons, and having every appearance of being perfectly good. In preparing these for use, two or three were dissolved in a small quantity of snow water, this solution being kept in a vial, which was carried in the pocket. A thread soaked in this was the medium of introduction.

From this solution twenty-eight persons were vaccinated on the day it was thus prepared (Feb. 11th), and no bad results followed. On the 18th, one week after, twenty-seven or eight others were vaccinated with the same material. Five of thes were seriously affected with constitutional symptoms, followed by violent erysipelas of the whole arm, both external and cellular, and sloughing. Three days later, two more persons were vaccinated from the same vial, both of whom have since died.

It was the opinion of nearly all present at the time of the report, that the cause of these results arose from the decomposition of the animal matter in the solution, and that to this and not to any inherent peculiarity in the matter, nor to the mode of application, were to be attributed the unlooked-for and dangerous results which have followed.

That the symptoms were due to some *change in the matter, subsequent to its preparation*, is evident from the fact that those first vaccinated exhibited nothing unusual, while the symptoms of blood-poisoning were most marked in those last inoculated with the virus.

That the matter had commenced to undergo the putrefactive change, was evident from the odor, which was extremely offensive, so that the opinion generally entertained is obviously the correct one, that the symptoms were due to the introduction of putrid animal matter, and cannot be attributable to anything in the matter itself as originally obtained, nor in any peculiar atmospheric condition, of which there was no evidence.—*Boston Med. and Surgical Journal.*

